# Value of Quantitative SPECT/CT Lymphoscintigraphy in Improving Sentinel Lymph Node Biopsy in Breast Cancer

**DOI:** 10.1155/2022/6483318

**Published:** 2022-03-28

**Authors:** Ting Luan, Yongqing Li, Qingwei Wu, Yan Wang, Zongwei Huo, Xiaohui Wang, Ligang Xing, Xiaorong Sun

**Affiliations:** ^1^Department of Graduate, Shandong First Medical University, Shandong Academy of Medical Sciences, Jinan, Shandong, China; ^2^Department of Nuclear Medicine, Shandong Cancer Hospital and Institute, Shandong First Medical University, Shandong Academy of Medical Sciences, Jinan, Shandong, China; ^3^Department of Breast Surgery, Shandong Cancer Hospital and Institute, Shandong First Medical University, Shandong Academy of Medical Sciences, Jinan, Shandong, China; ^4^Department of Radiation Oncology, Shandong Cancer Hospital and Institute, Shandong First Medical University, Shandong Academy of Medical Sciences, Jinan, Shandong, China

## Abstract

**Methods:**

We retrospectively enrolled breast cancer patients who underwent SPECT/CT prior to sentinel lymph node biopsy. Quantification of radiotracer uptake from SPECT/CT data was performed. A radioactivity count threshold (*R*_SPECT_) using SPECT/CT was calculated for detecting metastatic sentinel lymph nodes. To localize sentinel lymph nodes exactly, we compared the positions of sentinel lymph nodes localized using SPECT/CT with positions localized surgically using an intraoperative *γ*-probe.

**Results:**

491 patients were included, with a median of 3 sentinel lymph nodes/patient detected by the *γ*-probe and 2 sentinel lymph nodes/patient detected by SPECT/CT. As the number of sentinel lymph nodes visualized on SPECT/CT images, the metastasis incidence of lymph nodes in the ≤2 SLNs group was significantly higher than that in the >2 SLNs group (35% vs. 15%, *P* < 0.001). No metastasis was found in lymph nodes with *R*_SPECT_ ≤ 30% in the >2 SLNs group, and thus, 30% (157/526) of SPECT/CT-identified nodes would avoid unnecessary removal. The positions of sentinel lymph nodes localized by SPECT/CT and *γ*-probe were identical in 42% (39/93) of patients.

**Conclusions:**

Quantitative Tc-99 m SC SPECT/CT imaging has the potential to preoperatively locate sentinel lymph nodes and intraoperatively avoid unnecessary sentinel lymph node biopsy.

## 1. Introduction

According to the data on the global cancer burden updated in 2020, the incidence of female breast cancer has surpassed lung cancer and now ranks first in the world [1]. Axillary lymph node status (AS) is a crucial prognostic factor in breast cancer patients. Accurate lymph node staging can guide treatment that affects the overall survival of patients [2, 3]. Sentinel lymph node (SLN) biopsy (SLNB) has replaced axillary lymph node dissection (ALND) and become an effective method to evaluate AS for patients with clinically node-negative breast cancer [4, 5]. Although SLNB is associated with less postoperative complications [6, 7], 3.5%–10.9% of patients experience lymphoedema and arm numbness caused by excessive SLNs removed [8–10]. Furthermore, more than 70% of excised SLNs have been examined to be healthy [11], prolonging the operating time and increasing the pathology workload and medical expenses of patients.

SLNs in breast cancer are detected using a combination of radiotracer and blue dye [12]. Affected by the small size of radiotracer particles and the long interval time between radiotracer injection and operation, the range of SLN detection is 1–10/patient and the average number of SLNs is approximately 3.24/patient [13]. It is a common practice to continue removing radioactive lymph nodes until the count rates are ≤10% of the hottest nodes by intraoperative *γ*-probe. However, Schuman et al. found that processing the two hottest lymph nodes and suspicious nodes is sufficient for initial axillary staging of breast cancer [14]. Practically, an intraoperative *γ*-probe may not detect the hottest SLN successfully for two main reasons: (i) the node is very close to the injection site, obscured by scatter and (ii) the position of the node is too deep to locate. In such circumstances, single-photon emission computed tomography/computed tomography (SPECT/CT) imaging may have an additional value.

SPECT/CT technology has the potential for SLN localization and quantitative analysis of radioactive lymph nodes. Preoperative SLN SPECT/CT imaging provides accurate anatomical information of SLNs with lymphatic drainage into the axilla and extra-axillary regions, distinguishing false-positive results caused by nuclide contamination and lymphatic tortuosity and reducing the false-negative rate caused by patient obesity. It compensates for the limitation of the *γ*-probe in determining the exact anatomy of SLNs. Moreover, in a recent study, Kwak et al., simulating the common practice of removing SLNs with ex vivo count rate ≥10% of the hottest node detected by *γ*-probe, proposed the concept of the radioactivity count threshold, which was calculated based on SPECT/CT quantification, and found that no metastatic SLN (m-SLN) was detected when using a threshold of 20% [15]. Nevertheless, the value of quantitative SPECT/CT of SLNs in breast cancer is unknown.

This study aimed to investigate the feasibility of a radioactivity count threshold using ^99m^Tc-SC SPECT/CT quantification in helping surgeons select SLNs that are likely to be metastatic and minimize the number of SLNs removed. In order to help surgeons quickly locate SLNs, we evaluated the accuracy of ^99m^Tc-SC SPECT/CT for preoperatively marking the position of SLNs.

## 2. Materials and Methods

### 2.1. Patients Selected

Procedures performed in this study were approved by the institutional review board and ethics committee of Shandong Cancer Hospital. All consecutive breast cancer patients scheduled for SLNB were collected between December 2020 and June 2021. Inclusion criteria were primary breast cancer (T1-3) and no palpable lymph node at diagnosis, ≤2 suspicious nodes on imaging, or ≤2 positive nodes confirmed by needle biopsy ± clip placement. Patients with pregnancy and inflammatory breast cancer were excluded. Several patients were randomly selected for preoperative SLN localization on skin. Informed consent was obtained from each patient.

### 2.2. Imaging Protocol

A dual-head SPECT/CT scanner with low-energy high-resolution collimators (Discovery NM/CT 670 Pro, GE Healthcare, USA) was used. Each patient was given a peritumoral intradermal injection of 9.25 MBq (0.25 mCi) ±10% of ^99m^Tc-SC (Beijing Shihong Co. Ltd; Beijing, China) for surgery planned on the same day or twice the dose (0.5 mCi) for surgery arranged the next day. Planar scintigraphy (PS) was acquired after 20 min (within 4 h), after the injection of the radiotracer, with 3-minute static images in the anterior and lateral projections (matrix 256 × 256, zoom 1), followed by SPECT/CT (matrix 128 × 128, zoom 1) with the patient lying supine as well as arms positioned above the head. Images were processed by OSEM (ordered-subset expectation maximization) reconstruction and attenuation correction.

### 2.3. SLN Localization

We have made a simple tool for preoperative SLN localization. Ten metal sticks (diameter: 1.5 × 10^−3^ m, height: 0.24 m) were arranged at an interval of 0.03 m and fixed with tape orderly. The localization tool was attached to the side with cancer of the patient's chest closely before SPECT/CT. The radiologist diagnosed SLNs on SPECT/CT images and selected the hottest SLN for localization. The position of the hottest SLN was mutually determined by the localization tool, and a laser line of SPECT/CT was marked for surgery reference.

### 2.4. SLNB and Histopathology

SLNB was operated on the same day as the radiotracer injection or the next day in the morning. A peritumoral intradermal injection of 1% methylene blue (Jichuan Pharmaceutical Group Co. Ltd, Jiangsu, China) was performed 10–15 min before surgery. An intraoperative *γ*-probe (Neoprobe Corporation, AR-MED Ltd) was used for detecting radioactive nodes. Lymph nodes stained blue and/or ≥10% of the ex vivo counts of the hottest node were removed. Suspicious lymph nodes palpable by hand were also processed. Harvested nodes were sent for pathological examination. SLN status (hot/blue/suspicious), position, radioactivity counts, and pathological results were recorded.

### 2.5. SLN Quantification

**Figure 1:**
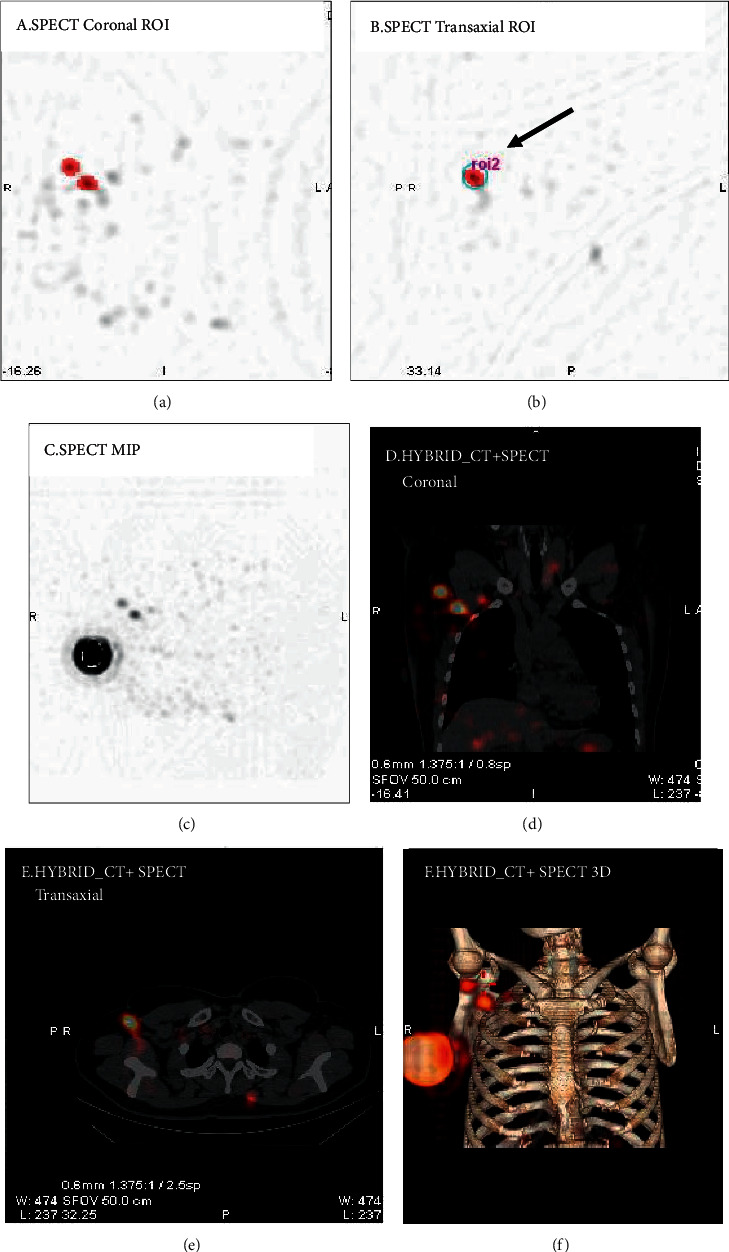
Graphical images describing the semiautomatic segmentation for the region of interest (ROI) and generation of radioactivity counts of hot nodes. (a–f) Coronal, transaxial, and MIP views of SPECT and corresponding hybrid SPECT/CT images.

Quantification of radiotracer uptake in the SLNs from SPECT/CT images was processed in the “Volumetrix MI Evolution for Tissue” module of the Xeleris Functional Imaging Workstation (Xeleris Version 4.0). The radiologist identified radioactive lymph nodes and chose semiautomatic segmentation for the region of interest (ROI) on the highest radioactive uptake SPECT layer. Radioactivity counts of SLNs are generated by clicking on the radioactivity concentration of each node. Graphical images describing the location of the ROIs and the generation of radioactivity counts of hot nodes on SPECT/CT are shown in Figure 1. The intraoperative *γ*-probe detected each extracted SLN for 10–15 s, and the final radioactivity count was obtained by pressing “target button.” The hottest radioactive node detected by either modality (SPECT/CT or *γ*-probe) was designated as 100%, and the following SLN was calculated as a percentage of the hottest node detected by SPECT/CT quantification or by *γ*-probe, expressing as *R*_SPECT_ and *R*_*γ*-probe_.

### 2.6. Statistical Analysis

Data analysis was processed using SPSS 19.0. Categorical variables of patient characteristics and metastasis incidence of SLNs were analyzed using the chi-square test or univariate Fisher exact test. Numerical variables were tested for normality and applied the Kruskal–Wallis test and two-sample *t*-test. A *P* value of <0.05 was considered as statistically significant.

## 3. Results

### 3.1. Clinical Characteristics and SLNB

491 consecutive breast cancer patients who underwent PS and ^99m^Tc-SC SPECT/CT prior to SLNB were enrolled. 1504 SLNs were excised using a combination of a radiotracer and blue dye, with a median of 3 SLNs/patient. SLNs were identified by SPECT/CT in 82% (404/491) of the patients, with a median of two SLNs/patient. Surgeons removed every node identified by SPECT/CT in 87% (350/404) of the patients. In the remaining patients, the same nodal level was sampled, but the number of SLNs removed intraoperatively was not equivalent to the number identified by SPECT/CT. The most common reason for this phenomenon was that no radioactivity was detected by the *γ*-probe at this location.

The clinical characteristics of patients are summarized in Table 1. Patients with early-stage breast cancer had more SLNs identified than those with locally advanced breast cancer, whether detected by SPECT/CT (*P* < 0.001) or by *γ*-probe (*P*=0.049). Additionally, patients younger than 50 y had more SLNs identified by SPECT/CT than those older than 50 y (average: 2.04 vs. 1.55, *P*=0.001). Patients with a history of tumor excisional biopsy tended to have more intraoperative SLNs (average: 3.50 vs. 3.02, *P*=0.046). Furthermore, we found that SLN metastasis rates were different in patients with different AJCC stages (*P* < 0.001) and were statistically higher in male breast cancer than female (75.0% vs. 23.6%, *P*=0.045).

### 3.2. Advantages of SPECT/CT Imaging over Planar Scintigraphy

PS visualized SLNs in 66% (322/491) patients and SPECT/CT visualized SLNs in 82% (404/491) of the patients, resulting in a 16% improvement in the visualization rate (*P* < 0.001). The most common region of lymph node drainage was the axilla, accounting for 94% (832/886) of all nodes. In 9% (37/404) of the patients, 33 and 11 lymph nodes drained to internal mammary and supraclavicular regions, respectively. 15 cases were presented as hot nodes on PS but interpreted as false-positive results due to tracer contamination on SPECT/CT images.

### 3.3. Relationship between the Number of SLNs Shown on SPECT/CT and Lymph Nodes Metastasis

Lymph nodes were visualized on SPECT/CT in 404 patients, and AS was metastatic in 28% (112/404) of them. Of the remaining patients, 34% (30/87) of the patients with no lymph nodes visualized on SPECT/CT had metastatic lymph nodes. Metastasis incidence of lymph nodes had a significant difference between different numbers of SLNs (Table S1). Based on the median number of SLNs visualized on SPECT/CT images, the patients were divided into two groups: ≤2 SLNs and >2 SLNs (Table 2). The metastasis incidence of AS in the ≤2 SLNs group was significantly higher than that in the >2 SLNs group (35% vs. 15%, *χ*2 = 21.3, *P* < 0.001).

### 3.4. Quantitative SPECT/CT Analysis Identifying Metastatic SLNs

**Figure 2:**
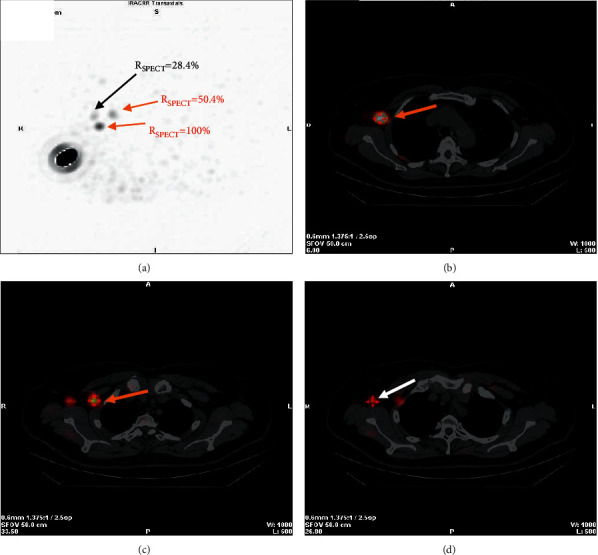
A 60-year-old female patient with invasive ductal carcinoma of the right breast. (a) Maximum intensity projection images. Three hot nodes are visualized with different *R*_SPECT_. Two nodes with higher *R*_SPECT_ (100%, 50.4%) are examined pathologically to be metastatic (red arrows), and another node with lower *R*_SPECT_ (28.4%) is healthy (black arrow). (b–d), SPECT/CT hybrid images for localizing the two metastatic nodes (red arrows) and the healthy node (white arrow). The surgeon might selectively avoid excising lymph nodes with lower radioactivity (*R*_SPECT_ ≤ 30%) deriving from preoperative SPECT/CT images.

In the >2 SLNs group, 15% (23/154) of patients were metastatic with 36 m-SLNs (6.8%, 36/526). To avoid removing invalid SLNs that were nonmetastatic, we analyzed the *R*_SPECT_ of m-SLNs in different 10% gradients (Table 3). Based on our data, no m-SLN was detected when *R*_SPECT_ ≤ 30% was used as a radioactivity count threshold. This would avoid resection of 30% (157/526) of the SPECT/CT-identified nodes, beneficial for 63 (40.9%) patients. A typical case describing the procedure of SLNB combined with SPECT/CT quantification is presented in Figure 2.

In 93 patients chosen for localization, radioactivity counts of SLNs were measured both by the *γ*-probe and SPECT/CT. A total of 29 m-SLNs were pathologically examined in 23 patients. The quantification of radioactive uptake in m-SLNs is listed in Table 4. The majority of m-SLNs occurred in the hottest nodes detected by SPECT/CT (79% (19/24)) or *γ*-probe (66% (19/29)). Four m-SLNs occurred in the second hottest nodes, and one m-SLN occurred in the third hottest node detected by SPECT/CT. Three m-SLNs mismatched with the SLNs presented on SPECT/CT images, but they were observed to have morphological abnormalities. The *γ*-probe found metastasis on a fifth hottest node. *R*_SPECT_ of m-SLNs were all over 30%, but no radioactivity count threshold of *R*_*γ*-probe_ could be found to identify the nature of the lymph node. When using the same radioactivity count threshold in the *R*_*γ*-probe_, we calculated a test sensitivity of 86% and 44.6% (139/312) of intraoperatively detected nodes that would not need to be removed.

### 3.5. SPECT/CT-Guided Intraoperative SLN Localization

93 patients were prospectively selected for SLN localization using SPECT/CT images. Positions of SLNs localized by SPECT/CT and by *γ*-probe were consistent in 42% (39/93) of the patients, and all were located in the anterior axillary region. In the remaining patients, the localization markers were located inside and/or below the position detected by *γ*-probe, with a deviation of 1–4 cm in distance. Particularly, a typical overweight patient, with a body mass index of 37 kg/m^2^, failed SLNB by using a combination of a radiotracer and blue dye. With the help of anatomic images and position markers localized by SPECT/CT, surgeons successfully removed a SLN in level I of the axilla, which was 6.69 cm away from the skin (Figure S1).

## 4. Discussion

In this study, we preliminarily developed a radioactivity count threshold for identifying m-SLNs using ^99m^Tc-SC SPECT/CT quantification, aiming to reduce invalid SLN removal for breast cancer. Patients with a history of prior excisional biopsy and younger than 50 y tended to have more SLNs detected either by a combination method or by SPECT/CT, implying that they would be the main beneficiaries of our findings. Additionally, it is feasible for advanced SPECT/CT to preoperatively locate SLNs.

It is well known that a combination of radioactive tracers and blue dyes increases the detection rate of SLNs, but it seems reasonable to believe that the removal of more nodes would lead to more adverse effects. Improving the accuracy of SLN detection is our objective. Numerous studies have focused on developing various novel radiotracers or hybrid approaches to improve SLN identification [16–19]; to the best of our knowledge, these methods have not been officially adopted in clinical settings because of their shortcomings. Less attention has been given to the evaluation of the relationship between radioactive uptake in metastatic and healthy SLNs.

Our study tried to investigate the value of quantitative SPECT/CT in distinguishing metastatic and healthy lymph nodes using ^99m^Tc-SC. ^99m^Tc-SC is the commonly used radiocolloid particle with a uniform size of 220 nm after filtration. It can be smoothly transported from the injection site to the SLNs but can cause excessive migration to secondary lymph nodes over time [20]. Based on this limitation, we hypothesized that metastases would more easily occur when fewer lymph nodes were visualized on SPECT/CT images owing to lymphatic duct obstruction by tumor cells, and distant lymph nodes with low tracer uptake affected by time extension would be less likely to metastasize.

As the number of SLNs shown on SPECT/CT images, we found that patients with ≤2 SLNs had significantly higher metastasis incidence than those with >2 SLNs. For radioactive uptake of SLNs, we developed a radioactivity count threshold that could guide selective removal of nodes by analyzing SPECT/CT quantification. When the radioactivity count threshold was set to 30% of the hottest nodes, no m-SLNs were missed, and 30% of the SPECT/CT-identified lymph nodes did not require resection. Our data preliminarily demonstrated that 99 m Tc-SC SPECT/CT has the potential to indicate AS and identify the nature of lymph nodes in breast cancer, assisting surgeons in judging whether lymph nodes could be monitored rather than excised in cases where the anatomic location of the node may be difficult for biopsy.

Clinical factors affecting the number of SLNs detected and metastasis incidence were also analyzed. Patients with younger age (≤50 y) and a history of prior excisional biopsy tended to have more SLNs detected, be exposed to unnecessary SLNB, and have more postoperative risks. These patients would be the target group of our findings. The m-SLNs more frequently occurred in male breast cancer patients than female patients (75% vs. 23.6%), which is consistent with previous studies [21]. Although male breast cancer is a relatively rare malignant cancer, accounting for less than 1.0% of all breast cancers, it has a worse prognosis than female breast cancer [22]. Therefore, the need for a thorough examination and pathological analysis of SLNs should be highlighted.

Preoperative SLN localization would reduce the scope of surgical resection, important for breast-conserving surgery. In a previous study, radioisotope (Co-57) was often appropriately positioned on the side of the body opposite the camera for reference, exposing patients to extra radiation [23]. We innovatively made a simple and safe tool for localizing SLNs. Regrettably, only 42% of the markers were completely consistent with the position determined by *γ*-probe. The remaining locations were all situated in a fixed range, with a deviation of 1–4 cm from the position using *γ*-probe. We believe that the posture difference between SPECT/CT imaging and surgery may be the main reason for this discrepancy. Due to the limitation of the small SPECT/CT aperture, patients lay supine with arms positioned above the head, whereas during SLNB, patients most often lay supine with arms extending perpendicular to the body. If possible, patients should maintain the same position during both SPECT/CT imaging and surgery. Our preliminary investigation of preoperatively marking of SLNs on skin according to SPECT/CT images is feasible and reliable, which would aid in probe-directed surgery and reduce operator-dependent variation and time, especially in obese patients.

Our study has several limitations. First, the detection rate of SPECT/CT alone was low (82%), compared with other studies (84.4%–92%) [24, 25]. There is a possibility that composition of the patients is different. Patients in previous research were strictly restrained to clinically node-negative. According to theNational Comprehensive Cancer Network (NCCN) guidelines version 4.2021 of breast cancer, SLNB indications are increasing. We enrolled a group of patients with ≤2 suspicious nodes on imaging or ≤2 positive nodes confirmed by needle biopsy, which is more suitable for the newest requirement clinically. Additionally, our preliminary findings need further validation in more patients from various hospitals and would be more meaningful when combined with recurrence of lymph nodes by follow-up, which we will take into account in the further study. Despite these limitations, we believe that our clinical procedures adhere to the NCCN guidelines and our findings are valuable for future research.

## 5. Conclusions

^99m^Tc-SC SPECT/CT quantification of SLNs might be helpful to guide selective radioactive node resection during SLNB and minimize unnecessary lymph node removal by establishing radioactivity count thresholds, especially for patients with prior excisional biopsy and younger than 50 y.

## Figures and Tables

**Table 1:** SPECT/CT and SLNB of 491 breast cancer patients.

Characteristic	Median (range)/freq (%)	Preoperative SPECT/CT		Intraoperative SLN detection by the combination method^*∗*^		SLN pathology	
		SLNs ($\bar{X}\pm s$)	Significance	SLNs ($\bar{X}\pm s$)	Significance	Patients with metastatic SLNs	Significance
Total	491					118 (24.0%)	
Age, y	50 (25–77)						
≤50	255 (51.9%)	2.04 ± 1.24	*P* < 0.001	3.15 ± 1.66	*P*=0.216	66 (25.9%)	*P*=0.319
＞50	236 (48.1%)	1.55 ± 1.36		2.97 ± 1.53		52 (22.0%)	
BMI, kg/m^2^	24 (16–37)						
＜24	223 (45.4%)	1.87 ± 1.34	*P*=0.285	3.17 ± 1.58	*P*=0.194	60 (26.9%)	*P*=0.174
≥24	268 (54.6%)	1.75 ± 1.29		2.98 ± 1.62		58 (21.6%)	
Gender							
Female	487 (99.2%)	1.80 ± 1.31	*P*=0.289	3.06 ± 1.60	*P*=0.389	115 (23.6%)	*P*=0.045
Male	4 (0.8%)	2.50 ± 1.73		3.75 ± 1.71		3 (75.0%)	
Primary tumor localization							
Upper outer quadrant	227 (46.2%)	1.78 ± 1.34	*P*=0.577	2.94 ± 1.64	*P*=0.052	57 (25.1%)	*P*=0.19
Upper inner quadrant	77 (15.7%)	1.78 ± 1.25		3.47 ± 1.29		25 (32.5%)	
Lower outer quadrant	61 (12.4%)	1.84 ± 1.43		3.03 ± 1.40		13 (21.3%)	
Lower inner quadrant	36 (7.3%)	2.08 ± 1.16		3.44 ± 2.04		8 (22.2%)	
Others	90 (18.3%)	1.74 ± 1.31		2.89 ± 1.61		15 (16.7%)	
Tumor size, cm	2(0.4–6)						
≤2	290 (59.1%)	1.81 ± 1.37	*P*=0.904	3.06 ± 1.54	*P*=0.940	61 (21.0%)	*P*=0.062
＞2	201 (40.9%)	1.80 ± 1.24		3.07 ± 1.68		57 (28.4%)	
AJCC clinical stage (v.8)							
0	18 (3.7%)	1.67 ± 1.33	*P*=0.001	2.78 ± 2.07	*P*=0.049	0 (0%)	*P* < 0.001
I	241 (49.1%)	1.92 ± 1.40		3.17 ± 1.42		32 (13.3%)	
II	203 (41.3%）	1.81 ± 1.22		3.07 ± 1.67		74 (36.5%)	
III	29 (5.9%)	0.86 ± 0.88		2.31 ± 2.00		12 (41.4%)	
Prior excisional biopsy							
Yes	48 (9.8%)	1.71 ± 1.24	*P*=0.595	3.50 ± 1.34	*P*=0.046	8 (16.7%)	*P* < 0.209
No	443 (90.2%)	1.81 ± 1.33		3.02 ± 1.62		110 (24.8%)	

^*∗*^Sentinel lymph nodes detected by the *γ*-probe and/or blue dye during sentinel lymph node biopsy.

**Table 2:** Comparison of metastasis incidence of axillary lymph nodes with a different number of SLNs by SPECT/CT.

No. of SLNs	Axillary lymph node status		Total	Metastasis incidence	*χ* ^2^	*P* value
	Metastasis	Nonmetastasis				
SPECT ≤ 2	119	218	337	35%	21.3	＜0.001
SPECT > 2	23	131	154	15%		

**Table 3:** *R*_SPECT_ of m-SLNs in the >2 SLNs group by SPECT/CT.

*R*_SPECT_	No. of visualized SLNs	No. of SLN metastases	Metastasis incidence
*R*_SPECT_ = 100%	154	17	11.0%
90% < *R*_SPECT_ < 100%	14	2	14.3%
80% < *R*_SPECT_ ≤ 90%	15	1	6.7%
70% < *R*_SPECT_ ≤ 80%	24	1	4.2%
60% < *R*_SPECT_ ≤ 70%	27	1	3.7%
50% < *R*_SPECT_ ≤ 60%	35	5	14.3%
40% < *R*_SPECT_ ≤ 50%	47	7	14.9%
30% < *R*_SPECT_ ≤ 40%	53	2	3.8%
20% < *R*_SPECT_ ≤ 30%	72	0	0
10% < *R*_SPECT_ ≤ 20%	55	0	0
0 < *R*_SPECT_ ≤ 10%	30	0	0
Total	526	36	6.8%

**Table 4:** Patients with metastatic sentinel lymph nodes (m-SLNs).

Patient no.	No. of SLNs by SPECT/CT	m-SLN location	m-SLN radioactivity		Blue dye	ALND
			*R*_SPECT_ (%)	*R*_*γ*-probe_ (%)		
1	1	L1	100	100	Negative	Yes
2	2	L1	100	100	Negative	No
3	2	L1	100	100	Negative	Yes
4	2	L1	100	100	Positive	Yes
4		L1	50^b^	56^b^	Negative	Yes
5	3	L1	100	100	Positive	Yes
6	2	L1	100	63^b^	Negative	No
7	1	L1	100	100	Positive	Yes
8	0	L1	Fail	100	Negative	Yes
9	1	L1	100	100	Positive	Yes
10	3	L1	100	100	Positive	Yes
11	2	L1	100	100	Positive	No
12	2	L1	100	100	Positive	Yes
12		L1	Not	83^b^	Positive	Yes
13	3	L1	100	100	Positive	Yes
13		L1	50^b^	49^b^	Positive	Yes
14	3	L1	97^b^	100	Positive	No
15	4	L1	51^b^	72^b^	Positive	Yes
15		L1	48^c^	41^c^	Positive	Yes
15		L1	Not	Not	Negative	Yes
16	0	L1	Fail	100	Negative	Yes
17	1	L1	100	100	Positive	Yes
18	2	L1	100	100	Positive	Yes
18		L1	Not	10^f^	Positive	Yes
19	1	L1	100	14^c^	Negative	Yes
20	2	L1	100	100	Positive	Yes
21	3	L1	100	100	Positive	Yes
22	1	L1	100	25^b^	Negative	Yes
23	3	L1	100	100	Positive	Yes

*R*_SPECT_, *R*_*γ*-probe_: the SLN was calculated as a percentage of the hottest node detected by SPECT/CT or *γ*-probe, and the hottest node detected by either modality was designated as 100%. ALND: axillary lymph node dissection. Fail: no lymph node detected on SPECT/CT. Not: palpable metastatic lymph nodes, which cannot be detected by SPECT/CT or *γ*-probe. b, c, f: second, third, fifth hottest node.

## Data Availability

The data used to support the findings of this study are available from the corresponding author upon request.

## Supplementary Materials

Supplementary Materials Figure S1: negative PS and positive SPECT/CT identification of hot node in 55-y-old overweight patient (BMI, 37 kg/m2). A, B, Anterior and left lateral PS showed no obvious hot uptake in the left axilla except injection site. C, CT slice showed an enlarged lymph node in the left axilla. D, SPECT/CT hybrid image showed a faint uptake was in concordance with the lymph node and 6.69 cm away from skin. Small metal shadow (white arrow) on hybrid images is our localization tool. Table S1: comparison of metastasis incidence of axillary lymph nodes with different number of SLNs by SPECT/CT.
