# Supplementary material for: Value of Quantitative SPECT/CT Lymphoscintigraphy in Improving Sentinel Lymph Node Biopsy in Breast Cancer
Source: Breast J. 2022 Mar 28;2022:6483318. doi: 10.1155/2022/6483318 (PMC9187279; doi:10.1155/2022/6483318)
Supplement: Supplementary Materials — Figure S1: negative PS and positive SPECT/CT identification of hot node in 55-y-old overweight patient (BMI, 37 kg/m2). A, B, Anterior and left lateral PS showed no obvious hot uptake in the left axilla except injection site. C, CT slice showed an enlarged lymph node in the left axilla. D, SPECT/CT hybrid image showed a faint uptake was in concordance with the lymph node and 6.69 cm away from skin. Small metal shadow (white arrow) on hybrid images is our localization tool. Table S1: comparison of metastasis incidence of axillary lymph nodes with different number of SLNs by SPECT/CT. [file 6483318.f1.docx]

**Supplementary Materials for**

**The Value of Quantitative SPECT/CT Lymphoscintigraphy in Guiding Sentinel Lymph Node Biopsy in Breast Cancer**


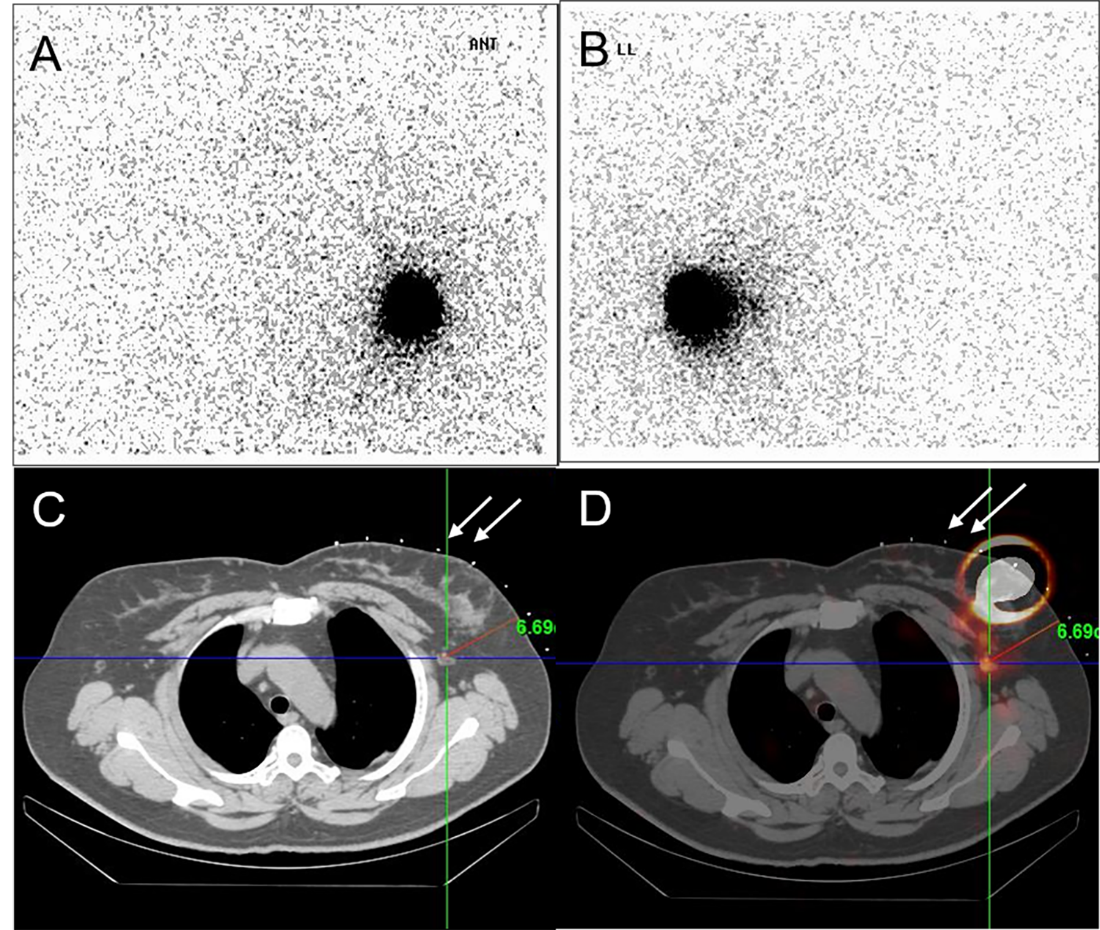


FIGURE S1. Negative PS and positive SPECT/CT identification of hot node in 55-y-old overweight patient (BMI, 37 kg/m^2^). A, B, Anterior and left lateral PS showed no obvious hot uptake in the left axilla except injection site. C, CT slice showed an enlarged lymph node in the left axilla. D, SPECT/CT hybrid image showed a faint uptake was in concordance with the lymph node and 6.69cm away from skin. Small metal shadow (white arrow) on hybrid images is our localization tool.

**TABLE S1**

**Comparison of metastasis incidence of axillary lymph nodes with different number of SLNs by SPECT/CT**

| NO. of SLNs | Axillary lymph node status | | Total | Metastasis incidence | | *χ^2^* | *P-*value |
| --- | --- | --- | --- | --- | --- | --- | --- |
|  | positive/patient | negative /patient |  |  |  |  |  |
| SPECT=0 | 30 | 57 | 87 | | 34% | 24.5 | ＜0.001 |
| SPECT=1 | 56 | 84 | 140 | | 40% |  |  |
| SPECT=2 | 33 | 77 | 110 | | 30% |  |  |
| SPECT=3 | 15 | 91 | 106 | | 14% |  |  |
| SPECT≥4 | 8 | 40 | 48 | | 17% |  |  |
